# A Rare Case of Sublingual Lipoma

**DOI:** 10.7759/cureus.61406

**Published:** 2024-05-31

**Authors:** Swapnil U Shinde, Jestina M John, Vishnuchandra Menon, Abdullah Tamboli, Kishan Dudhat, Mushtak Khan

**Affiliations:** 1 Department of Oral and Maxillofacial Surgery, Bharati Vidyapeeth (Deemed to be University) Dental College and Hospital, Sangli, IND

**Keywords:** floor of mouth, adipose tissue, wharton’s duct, rare tumors, benign tumors, clinical case report, sublingual lipoma

## Abstract

Lipomas are benign soft tissue tumors that are ubiquitous in nature. Available literature suggests that benign tumors are harmless unless they increase in size, resulting in compression of vital structures. This case report discusses the case of a 52-year-old man who presented to the clinic with a painless, growing lump on the right side of his mouth. The patient's symptoms included difficulty swallowing and speaking, which led the doctors to recommend surgical excision of the mass. There were no issues during the mass removal surgery, and the incision healed without compromising the lingual or hypoglossal nerves or Wharton's duct, as observed during follow-up visits. Patient history, symptoms, preoperative examination, treatment strategy, and surgical technique are all included in this case study, which focuses on the extremely unusual development of lipomas in the oral cavity, particularly on the floor of the mouth.

## Introduction

Lipomas represent the most prevalent benign neoplasms encountered within the human body, although their occurrence within the oral cavity is relatively infrequent. They are typically asymptomatic and are often discovered incidentally during clinical evaluation or radiographic imaging. However, when lipomas attain significant size, they may exert mechanical pressure on adjacent tissues, resulting in functional impairment. Surgical excision is commonly indicated to alleviate symptoms and mitigate the risk of malignant transformation. Lipomas, characterized by their asymptomatic nature and predominantly consisting of adipose tissue, typically demonstrate gradual development, primarily in the proximal extremities or trunk. While prevalent throughout the body, occurrences within the maxillofacial or oral regions are infrequent. According to epidemiological research, oral lipomas have an incidence rate of 1% to 4.4% [1]. The buccal mucosa accounts for approximately 45% of the most prevalent sites. Other important places include the lip, salivary glands, palate, and tongue. On the other hand, the floor of the mouth, or sublingual region, is a very uncommon location for lipomas [2].

Oral lipomas frequently localize to the buccal mucosa, typically manifesting as solitary submucosal or superficial nodules. These lesions commonly exhibit a yellowish hue or maintain the normal coloration of the surrounding mucosa. Their clinical presentation is often one of slowly expanding, painless masses that can be either soft or hard in texture. A painless, sessile, or pedunculated lump with a soft consistency and a protracted evolution period is the typical clinical presentation of intraoral lipomas (OLs). Yellowish nodules frequently appear as superficial lesions. The two most prevalent sites are the buccal mucosa and the tongue [3]. Lipomas can be classified based on their histological features, encapsulation, and invasion of nearby tissues. At 53.5%, benign adipose lesions in the mouth are simple lipomas, the most common. The complex structure of the maxillofacial region and the mouth cavity makes it difficult to treat lipomas, even though they are harmless and develop slowly. Their existence can cause problems due to a "mass effect," which involves compression or trapping of nearby muscles, glands, nerves, and dental structures [4], even though they usually do not act invasively. Impairments in processes, including mastication, speech articulation, and tongue movement, can result from these impacts on neighboring anatomy, which can become clinically substantial [5].

## Case presentation

**Figure 1:**
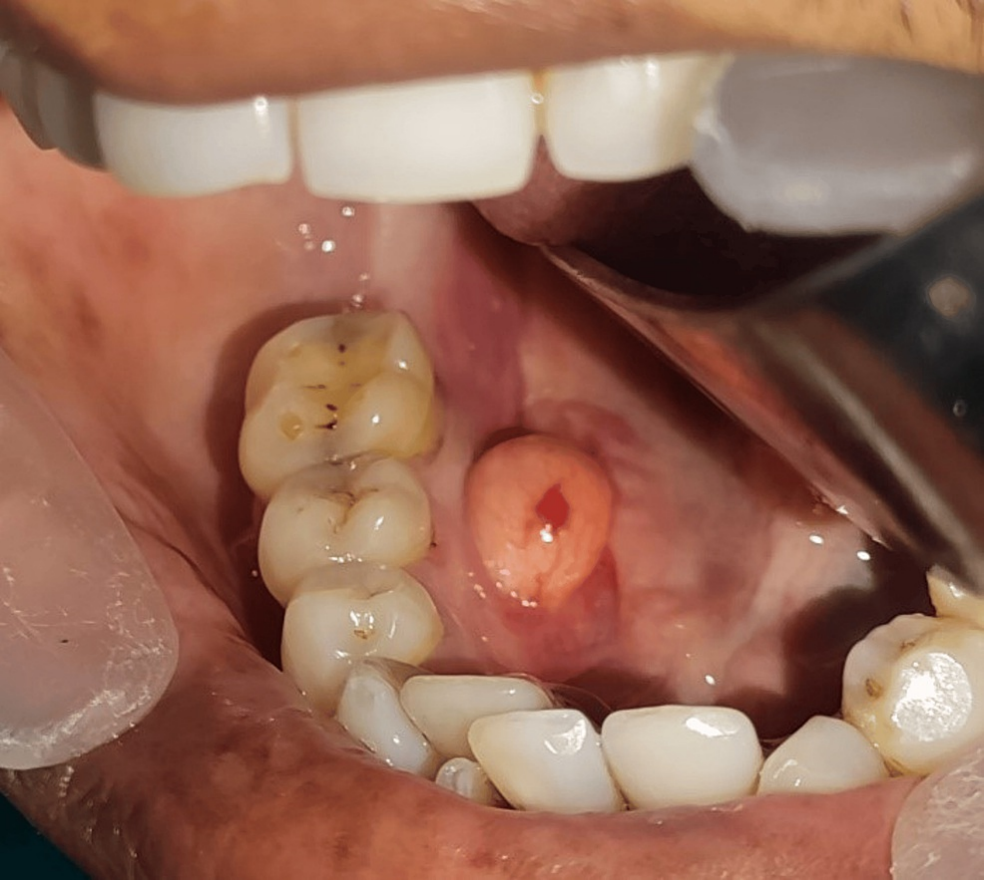
Intraoral swelling of the right floor of the mouth

A 52-year-old male patient presented to the outpatient Oral and Maxillofacial Surgery Department at Bharati Vidyapeeth Dental College and Hospital in Sangli with a complaint of an unidentified and painless mass on the right lingual side of the mandible near the floor of the mouth for six months. The swelling increased in size with time, and the patient began to experience pain when eating and speaking. He did not suffer from any systemic conditions. On examination of areas confined to teeth 45 and 46, an oval enlargement with a yellowish hue was noticed intraorally on the right side of the floor of the mouth (Figure 1).

**Figure 2:**
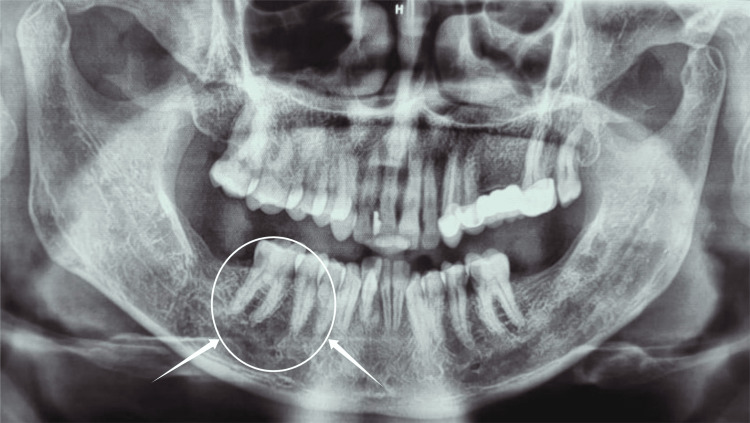
Orthopantomogram image: arrows showing no bone involvement in the regions of 45 and 46

The edema was found to be movable, firm, and non-tender upon examination. There were no signs of inflammation or ulceration on the surface mucosa. No bone involvement was found during the orthopantamograph (OPG)(Figure 2).

**Figure 3:**
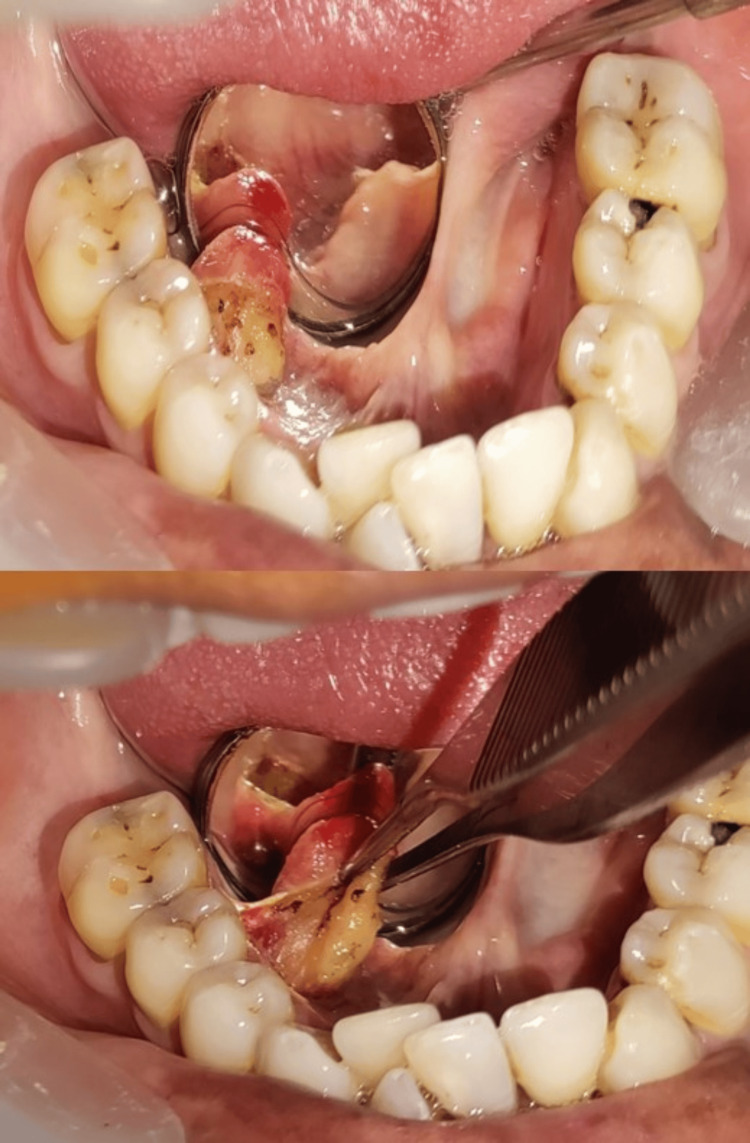
Excision of the specimen

A local anesthetic-induced excisional biopsy was scheduled. A pale pinkish-yellow mass that was uneven, poorly encapsulated, and lobulated was seen after blunt dissection, which had previously undermined the mucosal membrane. The specimen was excised and sent to the laboratory for histopathological evaluation (Figure 3).

**Figure 4:**
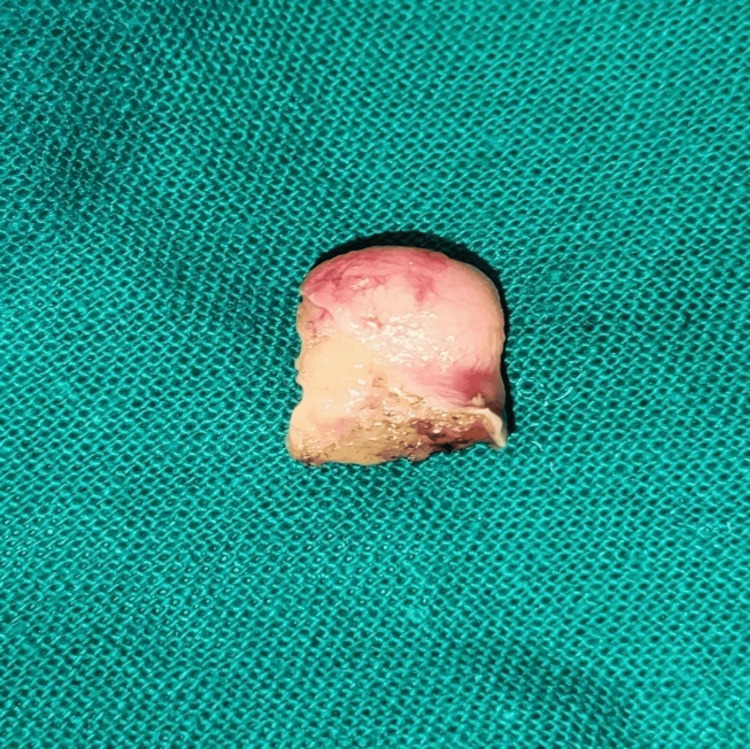
Excised specimen having a dimension of 2x1 cm

A histological analysis was conducted on the excised specimen, which had dimensions of 2×1 cm (Figure 4).

**Figure 5:**
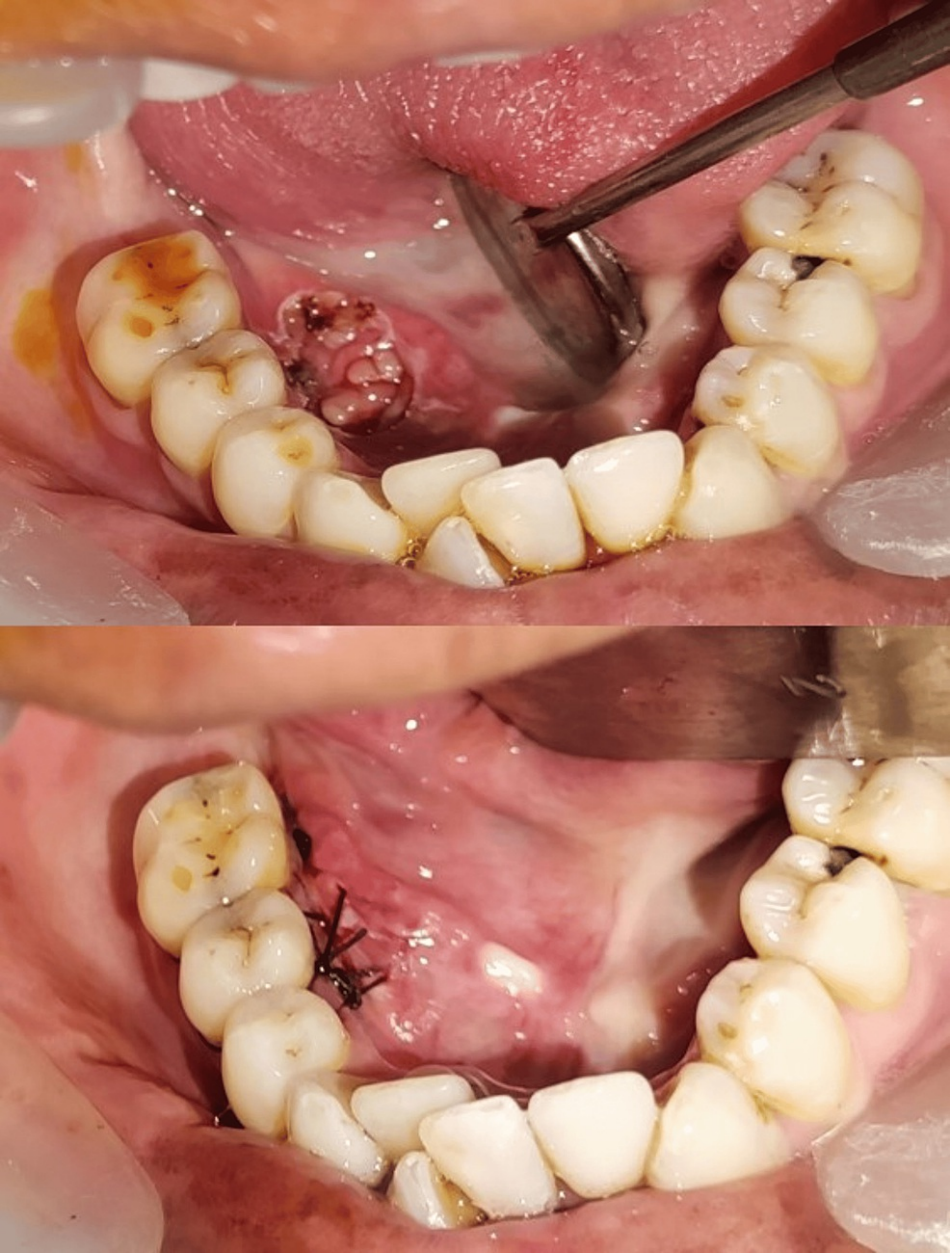
Sutures in place

Figure 5 presents the image of the area post-lesion removal and post-suture placement. Non-resorbable black braided silk 3-0 sutures were placed.

The histological examination revealed a stratified squamous epithelium and a fibrocellular connective tissue stroma. The stroma showed a well-circumscribed lesional tissue composed of mature fat cells enclosed within the fine areolar tissue surrounded by a fibrous capsule. The features of the histological examination were suggestive of "lipoma."

**Figure 6:**
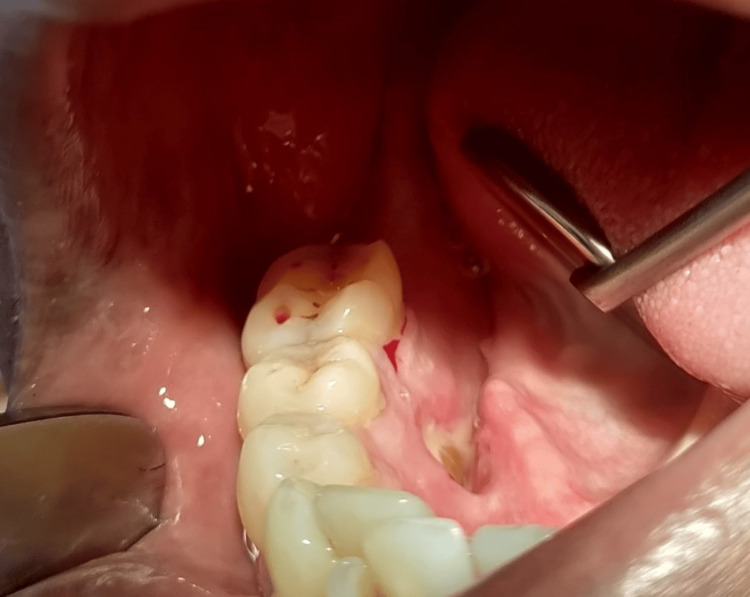
Follow-up image

The patient was prescribed a five-day antibiotic and analgesic course. On follow-up examination after seven days, the patient presented without any adverse events, and the operated site showed uneventful healing. Consequently, the sutures were removed (Figure 6).

## Discussion

When evaluating tumors on the floor of the mouth, it is important to do a thorough physical examination and explore a variety of possible diagnoses to determine the best course of treatment. Developmental, inflammatory, obstructive, and neoplastic causes are among the many possible explanations for these tumors. Dermoid cysts, lipomas, branchial cleft cysts, and thyroglossal duct cysts are typical developmental lesions seen in this area; the most common intraoral location for their presentation is the floor of the mouth [6]. Although there are several hypotheses, the exact cause of lipomas is still unknown. Lipomas may be linked to obesity and the uncontrolled expansion of fat cells, according to certain theories [7]. Possible embryonic sequestrations of multipotential cells triggered by adolescent hormones are the genesis of lipomas [8]. In addition, lipomas can occur in adipose tissue as a result of trauma or chronic inflammation.

Separating intraoral masses accurately requires a multifaceted strategy that includes taking a complete medical history, doing a full physical examination, imaging investigations, and pathological analysis. This example highlights the need to have a wide range of possible diagnoses to consider when finding an intraoral tumor, particularly in those who have smoked and had cancer in the past. Such a comprehensive approach ensures timely and appropriate therapy that is adapted to the unique clinical circumstances. This instance demonstrates, however, that lipomas that develop on the floor of the mouth might spread more widely. Dysphagia and dysarthria can be caused by large lipomas located on the floor of the mouth. It has the potential to induce hypoglossal nerve palsy in extreme instances [9].

Lipomas are defined histologically by the presence of adipose tissue enclosed in a fragile fibrous sheath. Subdivided further into classical/simple, adenolipoma, angiolipoma, chondroid, fibro-, myelolipoma, myolipoma, mycobiome, ossifying, sclerotic, and pericallosal lipomas, there are a great variety of lipomas under this categorization [4]. Histopathological analysis confirmed the presence of a typical lipoma in the presented instance; this subtype accounts for 45%-50% of all intraoral lipomas. Notably, fibrolipomas and typical lipomas are equally common on the floor of the mouth.

The primary method of treating lipomas is conservative surgical excision. Because of the rich neurovascular and glandular architecture in this location, the treatment presents inherent problems in instances involving large lesions located on the floor of the mouth. The nearby salivary glands, ducts, lingual and hypoglossal nerves, and relevant blood arteries must be carefully considered during surgical planning. A ranula can develop if the surgeon is not careful during the excision to avoid accidentally injuring the patient [10].

Neuromonitoring approaches have demonstrated great promise in reducing the likelihood of lingual nerve damage during operations involving the floor of the mouth during surgical procedures. Some surgeons are using neuromonitoring even though there are no established standards. Computerized tomography (CT) and magnetic resonance imaging (MRI) are two of the most important preoperative imaging modalities because they help with accurate diagnosis and surgical planning, which in turn improves the procedure's safety and effectiveness [11]. While research on the benefits of vagus and facial nerve monitoring after surgery is abundant, studies on hypoglossal nerve monitoring are scarce [7].

## Conclusions

Lipomas within the oral cavity, particularly on the floor of the mouth, are uncommon. These benign tumors rarely cause pain, although some patients may report mild discomfort. While it is rare, there is still a potential for these tumors to undergo malignant transformation. The standard treatment protocol involves surgical excision, which is a conservative approach to ensure complete removal and mitigate any risk of malignancy.
